# The effect of customer satisfaction on floral product purchase behavior, evidence from Shanghai, China

**DOI:** 10.1038/s41598-023-35137-0

**Published:** 2023-05-16

**Authors:** Shanshan Wang, Tinggui Chen, Chan Wang, Zengjin Liu, Lei Jia, Xintong Zhao

**Affiliations:** 1 College of Economics and Management, Shanghai Ocean University, Shanghai, 201306 China; 2 Institute of Agricultural Science and Technology Information, Shanghai Academy of Agricultural Sciences, Shanghai, 201403 China; 3 Graduate School of Agricultural Science Graduate, Tohoku University, Sendai, 9800845 Japan

**Keywords:** Software, Plant sciences

## Abstract

China's flower industry is developing rapidly, and the size of the retail market is increasing year by year. Studying the factors influencing residents' flower purchasing behavior and understanding their flower needs can help promote the sustainable development of the flower industry. Based on customer satisfaction theory, this paper uses 838 consumer research questionnaires from 15 districts in Shanghai to analyze the influence of customer satisfaction on residents' flower purchasing behavior by conducting a binary logit model and to investigate the moderating effect of flower purchasing purpose on the influence of satisfaction. The results show that price satisfaction and satisfaction with promotional methods have a significant negative effect on flower purchasing behavior, service satisfaction has a significant positive effect on purchasing behavior, and different customer purchase purposes lead to different intensities of the effect of satisfaction on purchasing behavior. According to the conclusion of the study, three countermeasures are proposed: to popularize the knowledge of flower culture, guide the concept of flower consumption, and promote the transformation of flower consumption to daily consumption; to conduct regular research on consumers by flower merchants to clarify consumers' needs and improve their satisfaction; to clarify consumers' purchase intention, increase the investment in the research and development and cultivation of flower products, and improve the supply level of flowers.

## Introduction

At present, the income level of households in China is increasing due to that fact that China’s economy is developing rapidly. The flower consumption is also beginning to change to mass consumption and daily consumption with people’s pursuit of high quality of life. And the consumption pattern is gradually personalized and diversified, the consumption range is developing from first- and second-tier cities to third- and fourth-tier cities and even the countryside, and the flower consumption group is growing. In recent years, China's flower industry has developed rapidly and the industrial chain tends to be complete. China has now become the world’s largest flower producer and important flower trading and consuming country. Date from the State Forestry and Grassland Administration show that China's flower plantation area in 2020 was 1,472,400 hectares with total sales of 20,061 million yuan, an increase of 60.46% and 134.41% compared with 2010. The total import and export trade of the flower industry in 2021 reached 701 million yuan, an increase of 12.66% compared with 2020. The main importers of Chinese flowers are the Netherlands, Japan, Ecuador, Thailand and Chile, and the main exporters are Japan, Korea, the Netherlands, the United States and Vietnam. China’s flower industry has initially formed a production layout of "fresh cut flowers in the southwest, seedlings and potted flowers in the southeast, seed bulbs in the northwest, and processed flowers in the northeast"^1^. The scale of the flower retail market is increasing year by year, and retail models such as offline purchase, live streaming, e-commerce, and community group purchase are jointly promoting the development of online and offline integration in the flower retail sector^2^. In addition, the construction of beautiful China in the 14th Five-Year Plan has put forward a huge demand for the flower industry. The continuous improvement of transportation conditions and infrastructure, increase in capital investmen have greatly promoted the development of China's flower industry. Vigorous development of the flower industry is the general trend. Shanghai is an international metropolis, Shanghai residents have a relatively strong desire and pursuit for a high quality of life, and the demand for floral products is also more vigorous. The Shanghai government also attaches great importance to the development of the flower industry and provided strong support for the rapid development of the flower industry. However, the focus on flower market demand and consumer behavior is not sufficient^3^. Therefore, in the context of vigorous development of the flower industry in Shanghai, this paper aims to study the influence of customer satisfaction on flower purchasing behavior based on consumer preference heterogeneity which is important for understanding customer needs, improving the products and services provided by the flower market, as well as promoting the sustainable and healthy development of the flower industry.

Consumer buying behavior has always been a topic of interest for scholars. In industry, it is also critical for enterprises to fully understand consumers' buying behavior, which is helpful to develop reasonable and effective sales strategies^4^. The purchase behavior of goods occurs because consumers derive satisfaction from the products and services provided^5^, and while consumers meet their own needs satisfied, they also consider whether there is an adverse impact on the environment and whether it will affect the needs of future generations^6^. Regarding consumer purchase behavior, Sheth et al.^7^ proposed the theory of consumer value, which suggests that five consumer values (functional, social, emotional, cognitive, and conditional values) influence whether or not consumers buy and which products they choose to purchase. Dalli & Romani^8^ argues that individual consumer behavior is the result of an intraindividual elaboration process that begins with the consumer's perception of an information stimulus, which the consumer stores in memory and use to shape his or her behavior to obtain a specific goal. With increased awareness of ecological conservation, consumers are more concerned about sustainability and green certification of products. Research in recent years has shown that product sustainability labels have important effects on consumers' ongoing purchase behavior^9^, and these effects vary by consumer and product type^10^. Consumers with higher environmental awareness are more willing to pay for green products and are also willing to pay more of a premium^11^. In addition, for individual products, such as insurance, organic food, and new energy vehicles, government economic policies, subsidy policies, and other policy elements can influence consumer purchasing behavior^12–14^. Consumers' values^15^ and emotional states^4^ towards different products can also have an impact on purchase behavior.

Plants are effective in reducing stress, easing emotions^16^, and improving quality of life, where flowers have an important role in satisfying residents' aesthetic needs^17^, conveying emotions^18^, such as expressing affection and gratitude. Therefore the reasons for choosing to buy flowers is mostly because of their aesthetic or emotional value^19^, such as beautifying the indoor environment, feeling nature^20^ or creating a good atmosphere in the home^21^. Numerous scholars have studied the factors influencing flower purchasing behavior, and Emanuele et al.^22^ analyzed the preferences of Italian flower consumers and showed that women buy flowers more frequently and that the frequency of flower purchases increases with age. For older people, material things become less important to them^23^ and they are more eager to find spiritual satisfaction in gardening-type activities^24,25^. Baourakis et al.^26^ conducted a study of Athens studied the flower market in Athens and concluded that age, income, and knowledge of flowers positively influence flower purchasing behavior. Poor flower quality and short flowering periods discourage consumers from purchasing flowers^27^. Most scholarly studies have shown that consumer demand for flowers increases significantly on holidays and anniversaries^28,29^, especially on Mother's Day and Valentine's Day.

Studying the factors that influence consumers' flower purchases is crucial to understanding flower demand^30^. Existing literature has mainly studied the influence of customers' characteristics such as gender and age as well as the characteristics of flowers on their flower purchasing behavior, but few studies have examined the influence of customer satisfaction on their purchasing behavior and lacked heterogeneity analysis of survey samples. Therefore, this study uses a binary logit model to analyze the effect of customer satisfaction on flower purchasing behavior based on 838 consumer research questionnaires from 15 districts in Shanghai and to investigate how the purpose of purchase affects the strength of the effect of satisfaction on purchasing behavior.

## Theroretical framework

### Customer satisfaction and purchasing behavior

Cardozo first introduced the concept of customer satisfaction into the field of marketing, and then several scholars studied satisfaction. Kotler^31^ believes that customer satisfaction is "a psychological state of pleasure or disappointment that is influenced by the difference between perceived effects and expectations", and Hunt^32^ describes customer satisfaction as "a test of expectations and an assessment of emotions". The current research on customer satisfaction mainly includes the influencing factors of customer satisfaction^33–35^, the evaluation system^36–38^, the impact on customer behavior and firm performance^39–41^, and some other scholars have studied the changes in customer satisfaction during COVID-19 in recent years^42–44^. There is a positive relationship between customer satisfaction and customers' attitude^45^, loyalty^46^, purchase intention and behavior^47–49^. The higher the customer satisfaction, the more positive the attitude toward the product and the more willing to buy the product. This helps to improve the performance of the company and reduce the cost of sales^50–52^, so the company must obtain customer satisfaction in order to gain long-term benefits^53^. The frequency and quantity of flowers purchased by consumers are influenced by variables such as consumers' perceptions and attitudes towards flowers^54^, and the frequency of flower purchases is significantly reduced when customers have negative attitudes towards flowers^55^, and the inability to meet consumer needs makes consumers dissatisfied with flower products, which also reduces the frequency of consumer purchases. In addition, although overall customer satisfaction with a product can predict customer behavior and intentions, it does not identify specific aspects to be improved^56^, therefore, this paper decomposes customers’ satisfaction with floral products and their purchase process into satisfaction with variety, quality, packaging, service, price, ease of purchase, and promotional methods, so that businesses can better improve their products and services to meet consumer needs. Accordingly, this paper argues that customer satisfaction will promote residents' flower purchasing behavior, the higher the satisfaction in each specific aspect, the more likely they are to purchase flowers frequently.

### Moderating role of purchase purpose

Customer satisfaction and buying behavior are not simply linear relationships, customers with the same satisfaction level will exhibit different buying behavior due to different personal characteristics^57–60^, in addition, to time and effort^61,62^, motivation and cognitive ability^63^ can also influence customers' buying behavior. The purpose of customers when purchasing goods or services plays a moderating role between customer evaluation and satisfaction^64^, purchase environment and purchase behavior^65^, product performance and repurchase behavior^66^, and satisfaction and behavioral intention^67^. Current research on the purpose of customer behavior is mainly focused on tourism. The study of the purpose of travel facilitates marketers to conduct market segmentation and develop more reasonable marketing plans to attract and retain customers^68–70^. Inspired by this, the differentiation and research on the purpose of customers' flower purchases is beneficial for flower producers and sellers to provide products and services that better meet customers' needs. Unlike traditional agri-food, the attributes of most flowers cannot be quantified, so the satisfaction consumers derive from consumption is closely related to the purpose of the purchase, which means that the demand for flowers is influenced by the purpose of consumers' flower purchases. Consumers who purchase flowers for gifting purposes are willing to pay more for fresh cut flowers at florists^71^ and pay more attention to the quality and packaging of flowers, while when flowers are purchased for personal use, consumers pay more attention to the variety, price, and color of the bouquet^26^. In this paper, the purchase purpose of flowers is divided into three types: gift, hobby, and decoration, where hobby means that customers love flowers and flowers can make them get spiritual satisfaction and relaxation, and decoration means that customers buy flowers to beautify their home environment and purify the air, etc. Accordingly, this paper argues that the purpose of consumers' flower purchases affects the strength of the role of satisfaction in flower purchase behavior.

**Figure 1:**
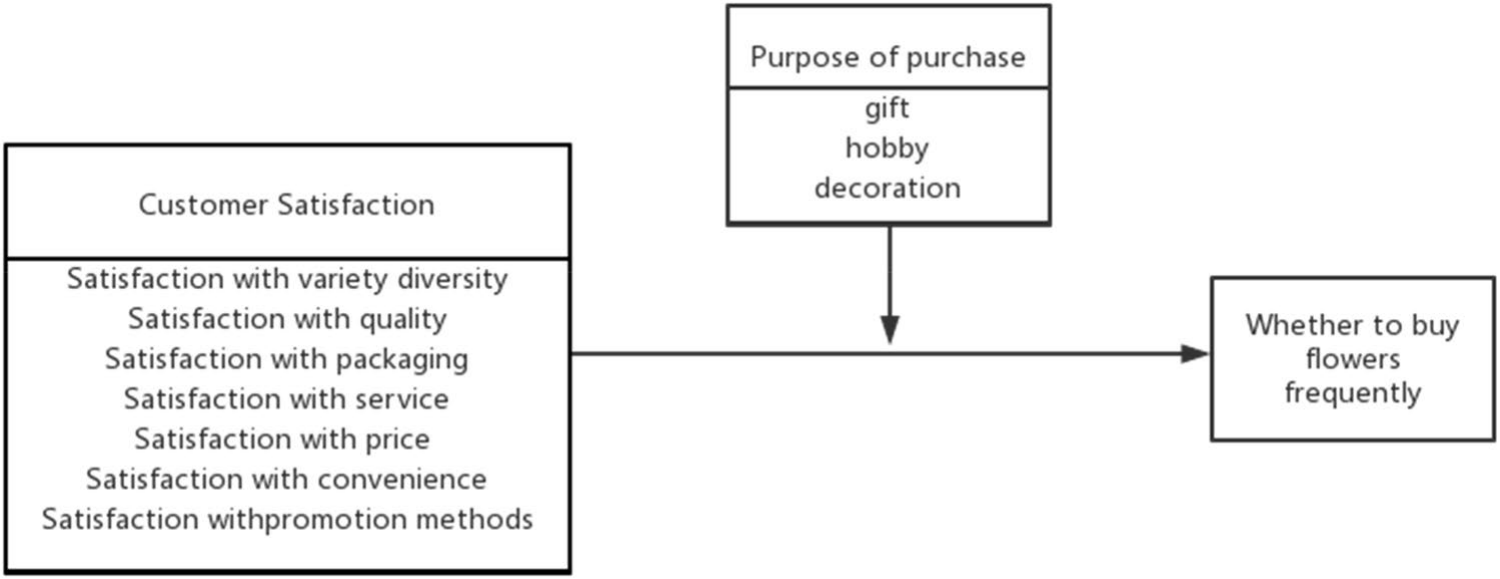
Hypothesized conceptual model.

In summary, the following analytical framework is constructed (Fig. 1). The framework indicates that customer satisfaction with flower variety diversity, quality, packaging, service, price, ease of purchase, and promotion methods directly affect customers' flower purchasing behavior, while the purpose of customers' flower purchase affects the strength of the effect of each satisfaction on flower purchasing behavior.

## Materials and methods

### Model settings

The explanatory variables in this study have only two choices, "yes" and "no", which is a typical dichotomous choice problem, so this study chooses a binary logit model to study it (which is free from the IIA property of the multinominal logit model). The IIA property is the ratio of the probability of choosing any two options for a given observation without being influenced by any other option, so the binary logic model can avoid the IIA restriction^72,73^. The model is constructed as follows:

1. Econometric model of the effect of customer satisfaction on flower purchasing behavior
  1 $$\mathrm{ln}\left[\frac{P\left({Y}_{i}=1\right)}{1-P\left({Y}_{i}=1\right)}\right]={\alpha }_{0}+\sum_{j=1}^{7}{\alpha }_{j}{X}_{ij}+{\alpha }_{8}{Z}_{i}+{\mu }_{i}$$
  In model (1), the ${Y}_{i}$ is the explained variable indicating whether the i-th consumer is a frequent buyer of flowers, and ${X}_{ij}$ (j = 1, 2……7) is the core explanatory variable indicating whether the i-th consumer is interested in variety diversity (${X}_{i1}$), quality (${X}_{i2}$), packaging (${X}_{i3}$), service (${X}_{i4}$), price (${X}_{i5}$), convenience (${X}_{i6}$), and promotion methods (${X}_{i7}$) in terms of satisfaction.${\alpha }_{j}$ (j = 1, 2……7) are the regression coefficients of ${X}_{ij}$, ${Z}_{i}$ is the control variable, and ${\alpha }_{8}$ is the regression coefficient of ${Z}_{i}$, the ${\alpha }_{0}$ is the constant term, and ${\mu }_{i}$ is the random error.
2. Econometric model of the role of purchase purpose in regulating the strength of customer satisfaction on purchase behavior
  2 $$\mathrm{ln}\left[\frac{P\left({Y}_{i}=1\right)}{1-P\left({Y}_{i}=1\right)}\right]={\alpha }_{0}+\sum_{j=1}^{7}{\alpha }_{j}{X}_{ij}+{\alpha }_{8}{Z}_{i}+{\alpha }_{9}{{G}_{ih}+\sum_{j=1}^{7}{\beta }_{j}{G}_{ih}{X}_{ij}+\mu_{i} } ,h=\mathrm{1,2},3$$
  Model (2) is based on model (1) with the addition of moderating variables ${G}_{ih}$ (h = 1, 2, 3), indicating that consumers' motives for purchasing flowers are gift (${G}_{i1}$), personal preference (${G}_{i2}$), decoration (${G}_{i3}$).${G}_{ih}{X}_{ij}$ denoting the interaction term between purchase purpose and each satisfaction, to analyze the difference in the strength of the role of different purchase purposes in moderating customer satisfaction on purchase behavior,${\alpha }_{9}$ and ${\beta }_{j}$ (j = 1, 2 …7) are the regression coefficients of ${G}_{ih}$ (h = 1, 2, 3) and ${G}_{ih}{X}_{ij}$ respectively.

### Measurement of variables

- *Explained variables* The explained variable in this study was "whether or not to buy flowers frequently", which was measured by the question "frequency of flower purchase", with the questionnaire options of "once a week" "2–3 times a week," "2–3 times a month," "once in 2–3 months," "holidays, anniversaries" "Rarely". "once a week" and "2–3 times a week" were defined as frequent purchases, while the rest of the purchases were considered infrequent. In this article, the term "frequent purchases" includes both online and offline purchases of flowers.
- *Core explanatory variables* The core explanatory variables of this study are consumer satisfaction with the characteristics of flowers, including "satisfaction with variety," "satisfaction with quality," "satisfaction with packaging," "satisfaction with service," "satisfaction with price," "satisfaction with convenience," and "satisfaction with promotion methods ", a total of seven different dimensions of satisfaction. The questionnaires are all measured on a five-point Likert scale, with the options of "very satisfied," "relatively satisfied," "average," "not very satisfied" and "very dissatisfied".
- *Adjustment variables* The consumers' "purchase purpose" was taken as the adjustment variable and measured by "what is the purpose of buying flowers". The questionnaire option was three discrete options: "gift", "hobby" and "decoration". Considering that the topic is a multiple choice, this study set it as three binary selection variables.
- *Control variables* The control variables in this study include respondents' characteristics and household characteristics, including gender, age, domicile, education, and occupation, and household characteristics including average monthly household income and the number of household members living together. Among them, the options of occupation in the questionnaire are "enterprise employees", "civil servants", "institutional employees", "self-employed private households ", "rural migrant workers", "jobless, unemployed or semi-unemployed", "students", "retired ", to maintain consistency with other variable types, they are divided into two categories: stable jobs and non-stable jobs, stable jobs include "enterprise employees", "civil servants", "employees of public institutions "The rest are non-stable jobs. The detailed description of each variable is shown in Table 1.

**Table 1:** Variable names and assignments.

Variables	Description of variables	Mean	SD
Explained variables	Whether to buy frequently (1 = yes, 0 = no)	0.21	0.41
Core explanatory variables	Species diversity satisfaction (1 = very satisfied, 2 = relatively satisfied, 3 = average, 4 = not very satisfied, 5 = very dissatisfied)	2.20	0.77
	Quality satisfaction (1 = very satisfied, 2 = relatively satisfied, 3 = average, 4 = not very satisfied, 5 = very dissatisfied)	2.27	0.74
	Packaging satisfaction (1 = very satisfied, 2 = relatively satisfied, 3 = average, 4 = not very satisfied, 5 = very dissatisfied)	2.31	0.73
	Service satisfaction (1 = very satisfied, 2 = relatively satisfied, 3 = average, 4 = not very satisfied, 5 = very dissatisfied)	2.35	0.74
	Price satisfaction (1 = very satisfied, 2 = relatively satisfied, 3 = average, 4 = not very satisfied, 5 = very dissatisfied)	2.57	0.75
	Convenience satisfaction (1 = very satisfied, 2 = relatively satisfied, 3 = average, 4 = not very satisfied, 5 = very dissatisfied)	2.38	0.80
	Promotion methods satisfaction (1 = very satisfied, 2 = relatively satisfied, 3 = average, 4 = not very satisfied, 5 = very dissatisfied)	2.57	0.80
Adjustment variables	Purpose of purchasing flowers		
	Gift (1 = yes, 0 = no)	0.26	0.44
	Hobby (1 = yes, 0 = no)	0.27	0.44
	Decoration (1 = yes, 0 = no)	0.45	0.50
Control variables	Gender (1 = male, 0 = female)	0.43	0.49
	Domicile (1 = local, 0 = foreign)	0.39	0.49
	Age (years)	38.23	13.78
	Education (1 = elementary school and below, 2 = junior high school 3 = secondary/high school, 4 = specialist, 5 = bachelor's degree 6 = graduate)	3.58	1.41
	Occupation (1 = enterprise and public service employees, 0 = other)	0.44	0.50
	Family population (number of people)	3.48	1.38
	Family monthly income (10,000 yuan)	1.68	1.40

### Date source

**Figure 2:**
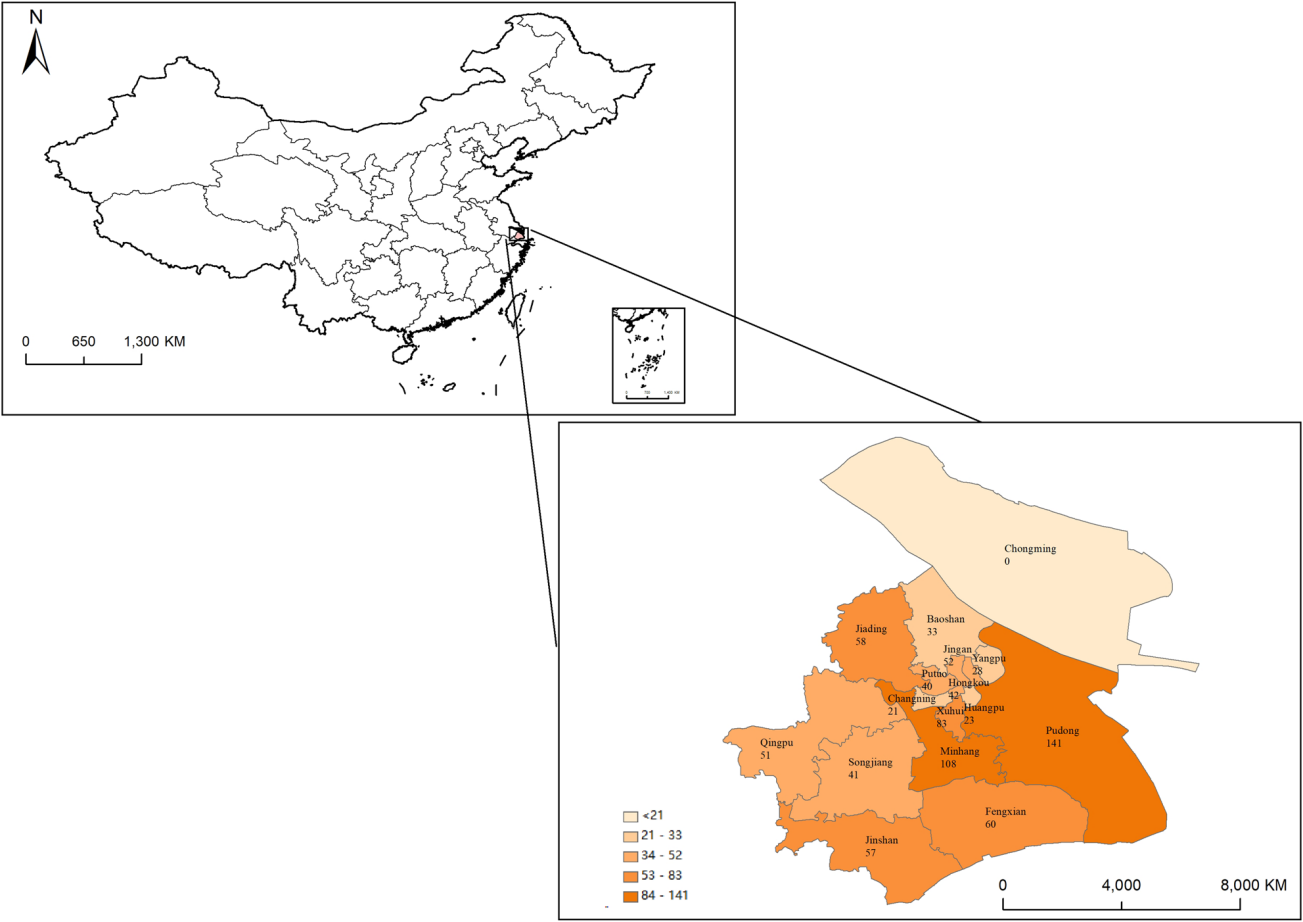
Sample size of each district.

All experimental protocols of this study were approved by the Science and Technology Commission of Shanghai, China, and all methods were performed in accordance with relevant guidelines and regulations, and informed consent was obtained from all participants before the questionnaire was administered. The data used in this study were mainly obtained from a field survey of flower consumers in 15 districts of Shanghai (except Chongming) in December 2019, and 838 valid questionnaires were finally obtained after the screening. To ensure the quality of the questionnaires, the surveyors were all postgraduate students with relevant training and pre-research, and a random sampling method was used to interview each respondent face-to-face. To make the obtained data more representative, the survey quantity was controlled according to the proportion of the resident population in each district of Shanghai, and the sample size and proportion of each district are shown in Fig. 2.

### Sample characteristics

The basic characteristics of this questionnaire can be divided into individual characteristics and household characteristics, where individual characteristics include gender, household registration, age, education, and occupation, and household characteristics include total household size and average monthly household income. The basic characteristics of the sample are described as shown in Table 2.

**Table 2:** Sample description.

Variables	Category	Frequency	Proportion (%)	Variables	Category	Frequency	Proportion (%)
Gender	Male	358	42.72	Career	Employees of enterprises and institutions, civil servants	370	44.15
	Female	480	57.28		Other	468	55.85
Domicile	Local	329	39.26	Family populati-on	1 person	58	6.92
	Field	509	60.74		2 people	130	15.51
Age	25 years old and below	161	19.21		3 people	286	34.13
	26–35 years old	271	32.34		4 people	163	19.45
	36–45 years old	160	19.09		5 or more	201	23.99
	46–55 years old	128	15.27	Family monthly income	Less than 5000 RMB	47	5.61
	55 years old and above	118	14.08		5000-9999RMB	198	23.63
Education	Elementary school and below	43	5.13		10,000-14999RMB	227	27.09
	Junior High School	190	22.67		15,000-19999RMB	82	9.79
	Junior High/High School	182	21.72		20,000 yuan and above	284	33.89
	Specialty	152	18.14				
	Undergraduate	203	24.22				
	Graduate Students	68	8.11				

In terms of gender, there were 358 males, accounting for 42.72% of the total sample, and 480 females, accounting for 57.28%. In terms of household registration distribution, there are 509 out-of-towners, accounting for 60.74% of the total sample. In terms of age, the most people were aged 26–35, accounting for 32.34% of the total sample, followed by those aged 26 and below, aged 36 to 45,aged 46 to 55, and aged 55 and above, accounting for 19.21%, 19.09%, 15.27% and 14.08% of the total sample, respectively. In terms of education, the number of respondents with elementary school education or less is the lowest, accounting for 5.13% of the total sample; 190 respondents have junior high school education, accounting for 22.67%; 182 respondents have secondary/high school education, accounting for 21.72%; and 423 respondents have college education or above, accounting for 50.47% of the total sample. In terms of occupation, the number of employees in enterprises and institutions, civil servants, and other occupations (self-employed private households, rural migrant workers, jobless, unemployed or semi-unemployed, and students) were not very different, each accounting for 44.15% and 55.85% of the total sample. In terms of the family population, 3-member families accounted for the largest share, 34.13%, and in terms of average monthly household income, 50.72% of the sample had an average monthly income between 5000 yuan and 15,000 yuan.

### Informed consent statement

Informed consent was obtained from all subjects involved in the study.

## Results and discussions

### Statistical analysis of consumer flower purchasing behavior

The survey results show that 84 respondents (10.02% of the total sample) buy flowers once a week, 91 respondents (10.86% of the total sample) buy flowers 2–3 times a week, 128 respondents (15.27% of the total sample) buy flowers 2–3 times a month, 212 respondents (25.30% of the total sample) buy flowers once every 2–3 months.The largest number of respondents chose to buy flowers on holidays or anniversaries, accounting for 26.49%, while the number of respondents who hardly bought flowers was 101, accounting for 12.05% of the total sample size.

Current Shanghai flower consumers are relatively satisfied with the flower market, as shown in Table 3. Compared to the price of flowers and promotional methods, consumers are more satisfied with the variety of flowers, quality, packaging, service, and ease of purchase, with more than half of them expressing satisfaction.

**Table 3:** Consumer satisfaction with flowers.

Type of satisfaction		Very satisfied	Relatively satisfied	Average	Not very satisfied	Very dissatisfied
Variety diversity	Frequency	138	427	247	19	7
	Percentage	16.47%	50.95%	29.47%	2.27%	0.84%
Quality	Frequency	116	407	292	18	5
	Percentage	13.84%	48.57%	34.84%	2.15%	0.60%
Packaging	Frequency	94	426	288	25	5
	Percentage	11.22%	50.84%	34.37%	2.98%	0.60%
Services	Frequency	96	386	329	21	6
	Percentage	11.46%	46.06%	39.26%	2.51%	0.72%
Price	Frequency	61	304	409	59	5
	Percentage	7.28%	36.28%	48.81%	7.04%	0.60%
Convenience	Frequency	104	369	314	45	6
	Percentage	12.41%	44.03%	37.47%	5.37%	0.72%
Promotion methods	Frequency	71	303	389	65	10
	Percentage	8.47%	36.16%	46.42%	7.76%	1.19%

### Analysis of the empirical results of the influence of customer satisfaction on flower purchasing behavior

To improve the accuracy of the estimation results,multicollinearity analysis was performed for model (1), and the variance inflation factors (VIFs) of each variable are shown in Table 4. The VIF values of each core explanatory and control variable are less than 10, indicating that there is no multicollinearity.

**Table 4:** Results of multicollinearity test.

Variable name	Quality satisfaction	Packaging satisfaction	Species diversity satisfaction	Service satisfaction	Convenience satisfaction	Promotion methods satisfaction	Price satisfaction	Control variables
VIF	2.19	1.99	1.96	1.92	1.73	1.67	1.63	< 10

Table 5 shows the empirical results of the effect of customer satisfaction on residents' flower purchasing behavior. Overall, price satisfaction (α_5_ = − 0.516, SE = 0.15, P < 0.01) and satisfaction with promotional methods (α_7_ = − 0.412, SE = 0.15, P < 0.01) have a significant negative effect on flower purchasing behavior, and service satisfaction (α_4_ = 0.296, SE = 0.17, P < 0.1) has a significant positive effect on purchasing behavior. Specifically, price satisfaction negatively affects flower purchasing behavior, the more satisfied customers are with the price of flowers, the greater the probability of purchasing flowers frequently. For consumers, price is something that is given up to obtain a certain product or service^74^, not only the objective price but more importantly the perceived price, the higher the satisfaction with the price, the more likely the customer is to purchase flowers more often as he/she believes that what he/she pays for flowers is worth it. Satisfaction with the promotion method negatively affects consumers' flower purchasing behavior, the more satisfied customers are with the promotion method of the merchant, the greater the probability of buying flowers more often. According to the stimulus-organism-response (SOR) model, merchants' promotional promotions are a powerful external stimulus for consumers, under which certain changes in their psychological state occur, such as stronger demand for flowers, which in turn causes an increase in their purchase frequency^75^. Service satisfaction has a significant positive effect on flower purchasing behavior, the more satisfied customers are with the merchant's service, the lower the probability of frequent purchases.

**Table 5:** Binary logit estimation of the effect of satisfaction on flower purchasing behavior.

	Logit	
	Whether to buy flowers regularly	
	Coefficient	Standard error
Species diversity satisfaction	0.164 (1.03)	0.16
Quality satisfaction	0.080 (0.47)	0.17
Packaging satisfaction	0.118 (0.70)	0.17
Service satisfaction	0.296* (1.77)	0.17
Price satisfaction	− 0.516*** (− 3.34)	0.15
Convenience satisfaction	0.030 (0.20)	0.15
Promotion methods satisfaction	− 0.412*** (− 2.84)	0.15
Gender	0.134 (0.75)	0.18
Domicile	0.403** (2.14)	0.19
Age	− 0.010 (− 1.36)	0.01
Education	− 0.205*** (− 2.78)	0.07
Occupation	− 0.115 (− 0.61)	0.19
Family population	− 0.014 (− 0.21)	0.06
Family monthly income	− 0.014 (− 0.21)	0.07
_cons	0.412 (0.65)	0.64
N	838	

*Indicates significant at the 10% level, ** indicates significant at the 5% level, *** indicates significant at the 1% level.

### Empirical analysis of the effect of purchase purpose moderating satisfaction on flower purchasing behavior

The core explanatory and moderating variables were regressed according to model (2) after centralized treatment, and Table 6 shows the empirical results of the strength of the role of purchase purpose in moderating customer satisfaction on flower purchase behavior. In general, when the purpose of buying flowers is to give them as gifts, the positive effect of satisfaction with the packaging on flower purchasing behavior is weakened (α_3_ = 0.118, β_13_ = − 1.604, SE = 0.53, P < 0.01), and the positive effect of satisfaction with ease of purchase on purchasing behavior is enhanced (α_6_ = 0.030, β_16_ = 0.732, SE = 0.42, P < 0.1). When the purpose of purchasing flowers is to satisfy personal hobbies, the positive effect of satisfaction with variety on flower purchasing behavior (α_1_ = 0.164, β_21_ = − 1.037, SE = 0.42, P < 0.05) and the negative effect of satisfaction with price are weakened (α_5_ = − 0.516, β_25_ = 0.836, SE = 0.37, P < 0.05). When flowers are purchased to decorate the environment, the positive effect of satisfaction with ease of purchase on purchasing behavior is weakened (α_6_ = 0.030, β_36_ = − 0.644, SE = 0.31, P < 0.05). Specifically, when flowers are bought as a gift, consumers pay more attention to the packaging of flowers, which enhances the negative influence on purchasing behavior, the more satisfied they are with the packaging, the higher the probability of buying flowers more often, while the positive influence of purchasing convenience on purchasing behavior is enhanced. The reason is that when buying flowers as a gift to give people, what consumers value most is whether the packaging is exquisite. The beautifully packaged gift can better convey the feelings of the giver. Different from giving flowers as a gift, in order to satisfy their love for flowers, consumers will be more inclined to the diversity of flowers, hoping to buy different varieties of flowers. At this time, consumers will reduce the consideration of price, resulting in the negative effect of price satisfaction on the flowers purchase behavior is weakened. When flowers are purchased to decorate the home environment, the positive effect of convenience satisfaction on the flowers purchase behavior is diminished, and customers are more likely to buy flowers more often because of the convenience of getting them, the reason being that flowers are more likely to wilt, and flowers used to decorate the home need to be replaced frequently, so easy access to flowers becomes more important to such consumers.

**Table 6:** Binary logit estimation of the effect of purchase purpose moderated satisfaction on flower purchasing behavior.

	Whether to buy flowers regularly					
	Coefficient	Standard error	Coefficient	Standard error	Coefficient	Standard error
Gift * species diversity satisfaction	0.122 (0.22)	0.55				
Gift * quality satisfaction	0.469 (0.85)	0.55				
Gift * packaging satisfaction	− 1.604*** (− 3.01)	0.53				
Gift * service satisfaction	− 0.078 (− 0.15)	0.50				
Gift * price satisfaction	− 0.374 (− 0.85)	0.44				
Gift * convenience satisfaction	0.732* (1.76)	0.42				
Gift * promotion methods satisfaction	− 0.315 (− 0.72)	0.43				
Hobby * species diversity satisfaction			− 1.037** (− 2.44)	0.42		
Hobby * quality satisfaction			0.001 (0.00)	0.42		
Hobby * packaging satisfaction			− 0.251 (− 0.63)	0.41		
Hobby * service satisfaction			− 0.147 (− 0.36)	0.42		
Hobby * price satisfaction			0.836** (2.26)	0.37		
Hobby * convenience satisfaction			− 0.179 (− 0.49)	0.36		
Hobby * promotion methods satisfaction			− 0.289 (− 0.79)	0.37		
Decoration * species diversity satisfaction					0.093 (0.29)	0.32
Decoration * quality satisfaction					− 0.106 (− 0.29)	0.36
Decoration * package satisfaction					0.105 (0.30)	0.35
Decoration * service satisfaction					0.246 (0.72)	0.34
Decoration * price satisfaction					− 0.096 (− 0.30)	0.32
Decoration * convenience satisfaction					− 0.644** (− 2.08)	0.31
Decoration * promotion methods satisfaction					− 0.060 (− 0.21)	0.30
_cons	− 0.503 (0.25)	0.54	− 0.616 (− 0.30)	0.55	− 0.416 (0.14)	0.53
N	838		838		838	

*Indicates significant at the 10% level, ** indicates significant at the 5% level, *** indicates significant at the 1% level.

In summary, purchase purpose affects the strength of the effect of customer satisfaction on flower purchasing behavior.

### Robustness testing

To verify the robustness of the empirical results, this study uses Probit regression to test the above results, and the results are shown in Tables 7 and 8. Both price satisfaction and satisfaction with promotional methods have a significant positive effect on flower purchasing behavior at the 1% level of significance, and service satisfaction has a significant negative effect on purchasing behavior at the 10% level of significance, and the purpose of purchase has a certain moderating effect. The results are consistent with the logit regression results, indicating that the results are robust.

**Table 7:** Probit estimation of the effect of satisfaction on flower purchasing behavior.

	Probit	
	Whether to buy flowers regularly	
	Coefficient	Standard error
Species diversity satisfaction	0.094 (1.05)	0.09
Quality satisfaction	0.047 (0.48)	0.10
Packaging satisfaction	0.066 (0.68)	0.10
Service satisfaction	0.167* (1.78)	0.09
Price satisfaction	− 0.294*** (− 3.34)	0.09
Convenience satisfaction	0.008 (0.10)	0.09
Promotion methods satisfaction	− 0.231*** (− 2.79)	0.08
Gender	0.066 (0.65)	0.10
Domicile	0.232** (2.13)	0.11
Age	− 0.006 (− 1.30)	0.00
Education	− 0.120*** (− 2.84)	0.04
Occupation	− 0.065 (− 0.60)	0.11
Family population	− 0.011 (− 0.30)	0.04
Family monthly income	− 0.008 (− 0.21)	0.04
_cons	0.222 (0.60)	0.37
N	838	

*Indicates significant at the 10% level, ** indicates significant at the 5% level, *** indicates significant at the 1% level.

**Table 8:** Probit estimates of the effect of purchase purpose moderated satisfaction on flower purchase behavior.

	Whether to buy flowers regularly					
	Coefficient	Standard error	Coefficient	Standard error	Coefficient	Standard error
Gift * species diversity satisfaction	0.025 (0.08)	0.31				
Gift * quality satisfaction	0.301 (0.99)	0.30				
Gift * packaging satisfaction	− 0.942*** (− 3.14)	0.30				
Gift * service satisfaction	− 0.051 (− 0.18)	0.28				
Gift * price satisfaction	− 0.231 (− 0.94)	0.25				
Gift * convenience satisfaction	0.426* (1.81)	0.24				
Gift * promotion methods satisfaction	− 0.142 (− 0.59)	0.24				
Hobby * species diversity satisfaction			− 0.612** (− 2.52)	− 2.52		
Hobby * quality satisfaction			− 0.011 (− 0.05)	− 0.05		
Hobby * packaging satisfaction			− 0.128 (− 0.56)	− 0.56		
Hobby * service satisfaction			− 0.120 (− 0.52)	− 0.52		
Hobby * price satisfaction			0.489** (2.31)	2.31		
Hobby * convenience satisfaction			− 0.064 (− 0.32)	− 0.32		
Hobby * promotion methods satisfaction			− 0.168 (− 0.82)	− 0.82		
Decoration * species diversity satisfaction					0.034 (0.19)	0.18
Decoration * quality satisfaction					− 0.043 (− 0.21)	0.20
Decoration * package satisfaction					0.039 (0.19)	0.20
Decoration * service satisfaction					0.152 (0.79)	0.19
Decoration * price satisfaction					− 0.030 (− 0.17)	0.18
Decoration * convenience satisfaction					− 0.373** (− 2.11)	0.18
Decoration * promotion methods satisfaction					− 0.042 (− 0.25)	0.17
_cons	0.094 (0.23)	0.43	− 0.113 (− 0.28)	0.41	0.040 (0.10)	0.41
N	838		838		838	

*Indicates significant at the 10% level, ** indicates significant at the 5% level, *** indicates significant at the 1% level.

## Conclusion and policy implications

This paper uses 838 consumer research questionnaires from 15 districts in Shanghai to empirically analyze the influence of customer satisfaction on residents' flower purchasing behavior and to investigate the moderating effect of flower purchasing purpose on satisfaction. The findings are as follows: first, price satisfaction and promotion satisfaction have a significant negative effect on flower purchasing behavior, while service satisfaction has a significant positive effect on purchasing behavior, so flower merchants should pay more attention to flower price and promotion. Existing research has found that the price of products and services has a positive impact on consumer purchasing behavior across a wide range of industries, including traditional industries such as food and apparel, as well as emerging online pre-sales and goods with special certification labels^76–78^. Although price is an objective external characteristic of a product, consumers usually perceive it as an external stimulus and process it to form their own subjective evaluations, which ultimately lead to consumer decisions. Consumers may infer the quality of a product from its price^79^, and if they believe that the quality of a product does not match its price, they may become dissatisfied with the price and thus influence their purchase behavior. This is consistent with the findings of this study. Product promotion mainly acts on consumers before the purchase behavior occurs, and consumer satisfaction with the promotional methods is mainly influenced by the information obtained^80^. A reasonable promotional approach will, to a certain extent, pull the change in consumer perceptions and stimulate consumer purchasing behavior^81^, which is consistent with the findings of this study. The reason why other satisfaction levels do not have a significant effect on residents' flower purchasing behavior may be that the transformation of customer satisfaction into purchasing behavior is also influenced by other factors. On the one hand, customer purchase decision is a complex and comprehensive process, and the conversion of customer satisfaction to purchase behavior is not 100%, but also influenced by product type, personal characteristics, purchase motivation and purchase purpose^57–63^. On the other hand, the accuracy of prediction of consumer behavior is related to the product purchased, and the prediction of purchase behavior for necessities is more accurate, while it is less accurate for other goods^82^.

Secondly, when flowers are purchased for the purpose of giving as a gift, the positive effect of satisfaction with the packaging on flower purchasing behavior is weakened, while the positive effect of satisfaction with ease of purchase on purchasing behavior is enhanced. When the purpose of purchasing flowers is to satisfy personal hobbies, the positive effect of satisfaction with variety on flower purchasing behavior and the negative effect of satisfaction with price are weakened. When flowers are purchased to decorate the environment, the positive effect of satisfaction with ease of purchase on purchasing behavior is weakened. This suggest that consumers value different floral product characteristics when they have different purchase purposes. The results of the above study were tested for robustness and still hold. Most scholars' studies show that consumers have different priorities when purchasing flowers when they are used for different purposes. For important days and occasions, consumers are more likely to buy flowers from a florist^26^, while when a consumer buys flowers for personal use, he/she is more likely to focus on the variety, price and color of the bouquet^55^. Huang^28^ believes that the two perceptions of "flowers are an everyday necessity" and "flowers are a gift" will influence the frequency of purchase. In addition, the purpose of purchasing flowers also affects the strength of the effect of store attributes on customer satisfaction.

The above findings are beneficial for flower producers and businesses to better understand the real needs of their customers, allowing them to integrate resources from all sides, increase research and development, and strengthen market construction from the customer's perspective, so as to provide flower products that better meet market demand and promote the sustainable development of the flower industry. It should be noted that the sustainable development mentioned in this paper is mainly about the impact on economic and social aspects, not only on the environment. The idea that "the concept of sustainability is only about the environment" is a "sustainability bias”^83^.

In general, China's flower industry is growing rapidly and the retail market size is increasing year by year. However, in recent years, due to the impact of COVID-19, the flower retail industry has seen a decrease in customers and a decline in retail sales, and both offline and online flower sellers have been affected to varying degrees. This phenomenon is not only seen in China, but also in other countries. In this paper, customer satisfaction is subdivided into six dimensions, and compared with existing literature, this subdivision is more useful to understand the detailed needs of customers and to identify specific aspects that need to be improved. In addition, by studying the moderating effect of purchase purpose, it is easier for flower producers and merchants to provide products and services that better meet customers' needs according to different scenarios, and drive customer spending. Based on the above research findings, this paper tentatively draws the following policy implications. First, popularizing the knowledge of flower culture and guide the concept of flower consumption. Among the 838 consumer research questionnaires, only 175 people frequently bought flowers, accounting for 20.88% of the total number, indicating that the flower consumption concept is not yet popular and residents' interest in flower consumption is still not high. The government is suggested to support and encourage flower production operators to hold various flower events in parks, scenic spots, and other places with high traffic flow according to the maturity time of different flowers. In addition, Carrying out small-scale flower arrangement experience activities and flower knowledge popularization activities to let people feel the beauty of flowers is also necessary. Secondly, putting consumer needs first and improving consumer satisfaction. Flower production operators should regularly conduct market research to keep track on the changing needs of different flower consumers; they are also suggested to make return visits to flower consumers and make appropriate adjustments based on consumer feedback. This can help them to make flower prices, types, quality, and services more in line with consumer needs, thereby improving consumer satisfaction. Third, clarifying consumers' purchase intentions and improve the level of flower supply. Flower production operators and wholesellers should pay more attention to whether flowers are beautifully packaged on holidays and special festivals, and adjust the supply of flower products appropriately according to customers' purchase intentions. At the same time, taking advantage of the Shanghai region, the government should increase investment in the research and development and cultivation of flower products, ensure the application of modern cultivation facilities and improve the quality and production of flowers.

## Data Availability

The questionnaires and datasets during the current study are not publicly available due to this study relies on the questionnaire results of the Shanghai government departments, but are available from the corresponding author on reasonable request. We have received the permission from appropriate authority to use the data.
