# Author Correction: The effect of customer satisfaction on floral product purchase behavior, evidence from Shanghai, China

**DOI:** 10.1038/s41598-023-37273-z

**Published:** 2023-06-20

**Authors:** Shanshan Wang, Tinggui Chen, Chan Wang, Zengjin Liu, Lei Jia, Xintong Zhao

**Affiliations:** 1 College of Economics and Management, Shanghai Ocean University, Shanghai, 201306 China; 2 Institute of Agricultural Science and Technology Information, Shanghai Academy of Agricultural Sciences, Shanghai, 201403 China; 3 Graduate School of Agricultural Science, Tohoku University, Sendai, 9800845 Japan

Correction to: *Scientific Reports* 10.1038/s41598-023-35137-0, published online 16 May 2023

The original version of this Article contained an error in Affiliation 3, which was incorrectly given as

‘Graduate School of Agricultural Science Graduate, Tohoku University, Sendai 9800845, Japan.’

The correct affiliation is listed below:

‘Graduate School of Agricultural Science, Tohoku University, Sendai 9800845, Japan.’

The original Article has been corrected.

